# Direct observation of finite size effects in chains of antiferromagnetically coupled spins

**DOI:** 10.1038/ncomms8061

**Published:** 2015-05-08

**Authors:** T. Guidi, B. Gillon, S. A. Mason, E. Garlatti, S. Carretta, P. Santini, A. Stunault, R. Caciuffo, J. van Slageren, B. Klemke, A. Cousson, G. A. Timco, R. E. P. Winpenny

**Affiliations:** 1 ISIS facility, Rutherford Appleton Laboratory, Chilton, Didcot, OX11 0QX Oxfordshire, UK; 2 Laboratoire Léon Brillouin CEA-CNRS, CEA Saclay, 91191 Gif-sur-Yvette Cedex, France; 3 Institut Laue-Langevin, 71 avenue des Martyrs, 38042 Grenoble Cedex 9, France; 4 Dipartimento di Fisica e Scienze della Terra, Università di Parma, Parco Area delle Scienze n.7/A, 43124 Parma, Italy; 5 European Commission, Joint Research Centre (JRC), Institute for Transuranium Elements (ITU), Postfach 2340, D-76125 Karlsruhe, Germany; 6 Institut für Physikalische Chemie, Universität Stuttgart, D-70569 Stuttgart, Germany; 7 Helmholtz Zentrum Berlin für Materialien und Energie, D-14109 Berlin, Germany; 8 School of Chemistry and Photon Science Institute, University of Manchester, M13 9PL Manchester, UK

## Abstract

Finite spin chains made of few magnetic ions are the ultimate-size structures that can be engineered to perform spin manipulations for quantum information devices. Their spin structure is expected to show finite size effects and its knowledge is of great importance both for fundamental physics and applications. Until now a direct and quantitative measurement of the spatial distribution of the magnetization of such small structures has not been achieved even with the most advanced microscopic techniques. Here we present measurements of the spin density distribution of a finite chain of eight spin-3/2 ions using polarized neutron diffraction. The data reveal edge effects that are a consequence of the finite size and of the parity of the chain and indicate a noncollinear spin arrangement. This is in contrast with the uniform spin distribution observed in the parent closed chain and the collinear arrangement in odd-open chains.

## Abstract (web summary)

Molecular magnets are among the smallest structures that may be exploited for quantum information processing. Here, Guidi *et al*. use polarized neutron scattering to observe finite size effects and a noncollinear spin arrangement in a Cr_8_Cd ring molecule, an even-numbered open antiferromagnetic spin-3/2 chain.

## Graphical abstract

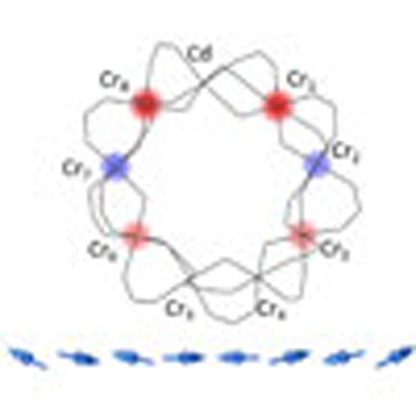

Antiferromagnetic (AF) Heisenberg spin chains have received intense theoretical and experimental attention since topological effects were found to affect the excitation spectrum and spin wave functions of the system^1^,^2^,^3^. For instance, edge states are predicted to exist in finite one-dimensional Heisenberg chains of *s*=1 spins and have been experimentally found through electron spin resonance measurements^4^,^5^ and inelastic neutron scattering^6^ in doped Haldane *s*=1 chains. Qin *et al*.^7^ have predicted that edge states also exist in half-integer (*s*>1/2) open AF chains with a staggering of magnetization along the chain.

Besides the fundamental interest in studying finite chain effects in the static and dynamical magnetic properties of AF chains, more recently finite chains have become the subject of an intense research effort in view of their potential application in the field of spintronics and quantum computation^8^,^9^,^10^. As finite AF chains are expected to show different magnetic behaviour depending on their length and on their symmetry properties^11^,^12^,^13^, their accurate experimental characterization is of crucial importance for controlling and manipulating their magnetic and quantum states. For example, Lounis *et al*.^11^ predicted that the ground state of chains of atoms deposited on a ferromagnetic substrate (equivalent to an applied external magnetic field) is very sensitive to the number of atoms in the chain. Even-numbered AF finite chains are expected to show a noncollinear (NC) spin structure while, below a critical length *L*_C_, odd-atom chains are predicted to show a collinear ferrimagnetic spin structure. Politi *et al*.^14^ have used a two-dimensional map method to extend the study to closed chains of atoms, finding that the odd-even effect persists in closed chains, with spins in even chains arranging themselves in a spin-flop (SF) state while a NC state exists in odd chains.

The experimental study of finite chain effects has traditionally involved a top–down approach where an ideal infinite chain is ‘cut' into shorter chains by introducing diamagnetic impurities. This procedure leads to an ensemble of chains with different lengths and different parities. Therefore any specific effect associated with the parity of the chain is averaged out in the experimental measurements. More recently the advances in building short chains of atoms using the capabilities of scanning tunnelling microscope tips to perform atom-by-atom manipulations^9^,^10^,^15^ has opened the possibility of creating more controlled short chain systems and finite size effects have been investigated experimentally^8^,^9^,^16^,^17^. However, all the experimental measurements carried out so far on such finite chains have provided only qualitative observation of the local magnetic moments and no quantitative values have been extracted. The quantitative understanding of the spin structure of nanomagnets is very important in the design of supramolecular chains for quantum information processing.

A more flexible alternative within the bottom-up approach is offered by the remarkable tunability and level of control reached in the chemistry of magnetic molecules. Advances in coordination chemistry achieved in the past 20 years allow the engineering of open or closed chains of atoms, with even or odd numbers of magnetic ions of integer or half-integer spins allowing the exploration of a host of different scenarios^18^,^19^. AF homometallic molecular rings are model systems to investigate the magnetic properties of chains^20^, as their periodic boundary conditions allow one to link their properties to infinite AF chains. On the other hand, the introduction of a non-magnetic impurity in the homometallic ring breaks the cyclic symmetry with an open-boundary condition resulting in an effective model system for an open chain. In addition, the molecules in the crystal are all identical allowing unequivocal probing of the behaviour of a specific system.

In this work we report the direct investigation of the local magnetic properties of an effective even-open chain of AF-coupled half-integer (3/2) spins in the field-induced (*S*=1) and (*S*=2) ground states using polarized neutron diffraction (PND). The system we chose to investigate, the molecular wheel Cr_8_Cd (ref. 21), provides a model of a finite Heisenberg AF open chain of spins 3/2 carried by the eight Cr^3+^ ions in the ring, which is interrupted by a diamagnetic Cd^2+^ ion. To date, there have not been any quantitative measurements of local magnetic moments of such a model system. Furthermore, we report analogous measurements on the parent even-closed ring Cr_8_ and compare the results with those obtained for Cr_8_Cd.

## Results

### Spin Hamiltonian and analysis of magnetic properties

**Figure 1:**
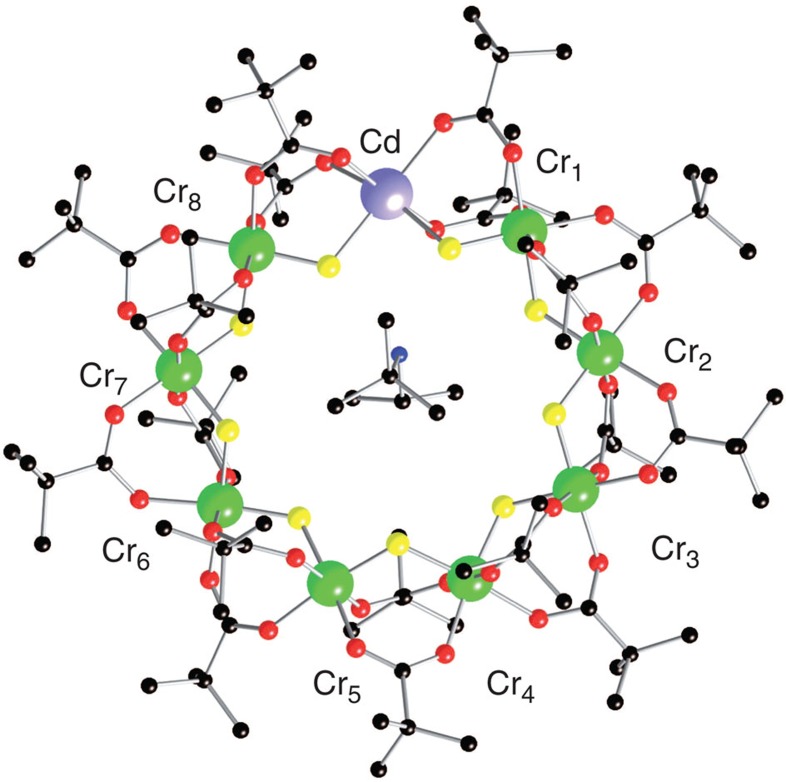
Structure of the Cr_8_Cd molecular ring. Cr atoms are represented in green, Cd in purple, O in red, F in yellow and C in black. Hydrogen ions are not shown for simplicity.

Single crystals of [H_2_N^*t*^Bu^*is*^Pr][Cr_8_CdF_9_(O_2_CCMe_3_)_18_] (Fig. 1) were synthesised following the procedure in ref. 21. From the crystal structure the Cd position is found to be ordered, with the Cd attached to the F-bridges that H-bond to the protonated amine. AF coupling between first nearest-neighbour Cr^3+^ ions, via the bridging organic ligands, leads to a singlet spin ground state *S*=0 and to excited states with integer-spin values *S*=1,2,... The energy-level diagram of the molecule has been investigated using multiple techniques, namely inelastic neutron scattering on powder samples^22^, high-field magnetization at low temperatures^23^ and muon spin relaxation^24^. The spin dynamics of each Cr_8_Cd molecule can be described by the Hamiltonian:


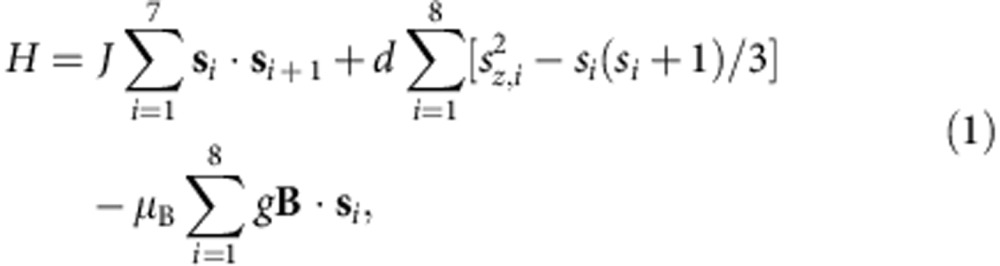


where **s**_*i*_ is the spin operator for the *i*^th^ ion in the molecule. The first term is the dominant nearest-neighbour isotropic Heisenberg exchange interaction. The second term describes a small uniaxial single-ion anisotropy (with the *z* axis perpendicular to the ring plane), while the last term is the Zeeman coupling with an external field **B**. INS measurements on the parent Cr_8_Zn compound^22^ and magnetization measurements on Cr_8_Cd (ref. 23) yield *J*=1.32 meV, *d*=−0.036 meV and *g*=1.98. The spin Hamiltonian parameters for the Cr_8_ ([Cr_8_F_8_Piv_16_], HPiv=pivalic acid) parent closed ring have also been accurately determined through INS studies^25^,^26^, which give *J*=1.46 meV and *d*=−0.038 meV (*g*=1.98).

**Figure 2:**
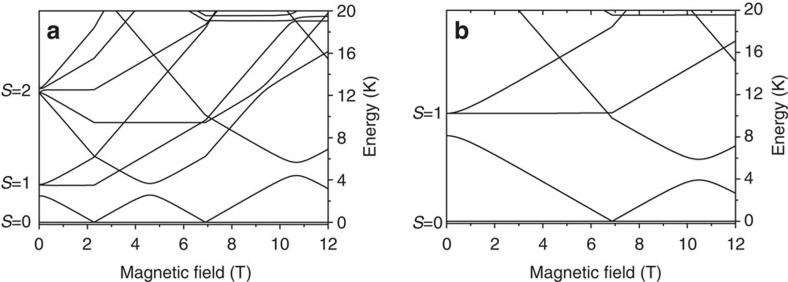
Energy-level diagrams as a function of magnetic field. Calculated energy-level diagram of Cr_8_Cd (**a**) and Cr_8_ (**b**) as a function of external magnetic field. Calculations are based on the spin Hamiltonian in Equation 1 with the field making an angle of 75.5° for Cr_8_Cd and of 90° for Cr_8_ with respect to the axis perpendicular to the rings.

Qualitative differences have been found in the energy-level diagram of Cr_8_Cd with respect to the parent Cr_8_ closed ring, in particular the energy of the first excited state |*S*=1, *M*_*S*_=0〉 is lowered from 0.7 meV in Cr_8_ to 0.2 meV in Cr_8_Cd, in spite of a very similar exchange coupling constant *J* (see Fig. 2). This can be qualitatively understood if we consider that it requires less energy to rotate the two spins at the end of an open chain as they only have one nearest-neighbour exchange interaction as compared with the two neighbours in the closed chain. This is also reflected in the magnetization measurements. The magnetization curves as a function of applied magnetic field at 0.15 K for Cr_8_ and Cr_8_Cd display characteristic steps, which correspond to the different spin ground states induced by the magnetic field^23^. The magnetic field necessary to induce a non-zero spin ground state in the Cr_8_Cd cluster is 2.3 T (see Fig. 2a), which is much lower than the 6.9 T needed for the Cr_8_ ring (Fig. 2b).

### Neutron diffraction experiments

**Figure 3:**
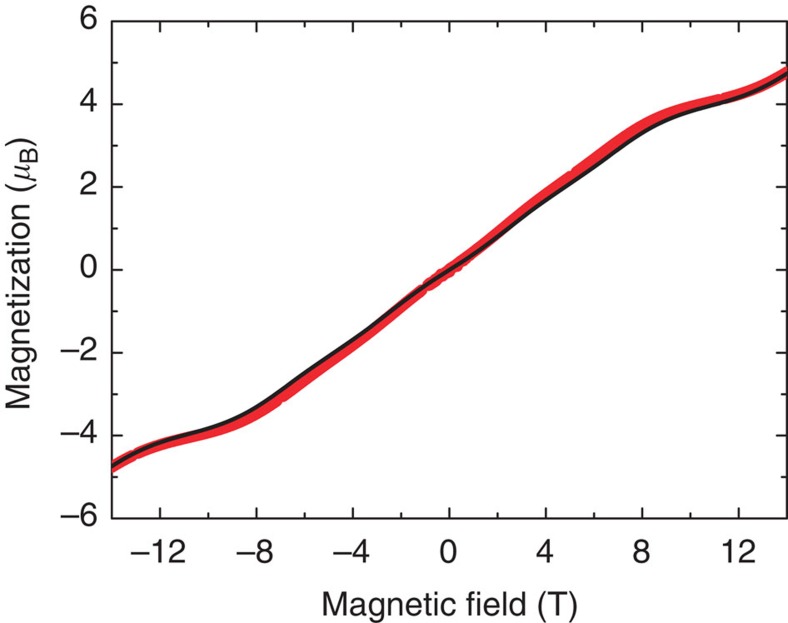
Low-temperature magnetization curve for Cr_8_Cd. Measured (red) and calculated (black) magnetization of Cr_8_Cd as a function of magnetic field for *T*=1.8 K and field along the crystallographic *a* axis. The field makes an angle of 75.5° with respect to the axis perpendicular to the rings.

**Figure 4:**
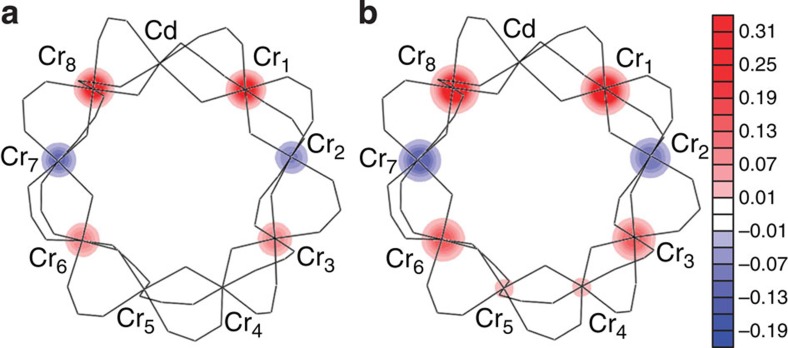
Measured spin density maps. Spin density maps (scale in *μ*_B_ Å^−2^) obtained by the refinement of the D3 experimental data for applied fields of 4.6 T (**a**) and 9 T (**b**) at *T*=1.8 K (projection along the crystallographic *b* axis).

The different topology of the two ‘open' and ‘closed' rings is expected to have an effect on the local spin moment distribution^7^,^11^,^14^. The theoretical calculations based on the quantum spin Hamiltonian in Equation 1 predict in Cr_8_Cd a non-uniform and staggered distribution of the spin moments along the chain, with an accumulation of magnetic moments at the extremities and a decrease of the spin moments with increasing distance from the Cd ion. To verify this prediction, we used PND for determining the experimental spin distribution in the heterometallic wheel Cr_8_Cd in non-zero spin ground states induced by applying a magnetic field at low temperature. PND was previously applied to the study of the magnetization density in molecular clusters like Mn_12_, Mn_10_, Fe_8_ (refs 27, 28, 29, 30) in their high-spin ground state to determine the arrangement of the magnetic moments on the metal ions of the cluster. Further, a magnetically driven non-zero spin ground state was studied by PND in the case of an organic magnetic tetramer^31^. The complexity of the PND experiment is rewarded by the unique possibility of measuring with great accuracy the distribution and magnitude of the local spin density, which is not yet achievable with other techniques. For a spin density PND study, it is required to first determine the nuclear structure factors at low temperature and therefore a neutron structural study at 15 K was performed before the PND experiment using the D19 diffractometer at the Institut Laue-Langevin (ILL). The unit cell and space group assumed were based on the original structure determined at 100 K by X-ray diffraction (ref. 21); the Cr_8_Cd compound crystallizes in the monoclinic P2_1_/n space group, with *a*=19.3987(8) Å, *b*=22.2606(8) Å, *c*=31.3094(14) Å, *β*=91.663(4) degrees and *Z*=4. The neighbouring Cr^3+^ and Cd^2+^ ions are connected by two pivalate groups -OCC(Me)_3_O- and one F atom, forming the nearly planar Cr_8_Cd wheel displayed in Fig. 1. In the neutron diffraction data at low temperature, weak extra reflections were observed between the peak positions corresponding to the P2_1_/n space group indicating a structural change (see Supplementary Note 1 and Supplementary Table 1 for details). PND measurements where performed on the D3 diffractometer at ILL with a magnetic field applied along the crystallographic *a* axis. With an applied magnetic field of 4.6 T and *T*=1.8 K the system is driven to a spin ground state of |*S*=1, *M*_*S*_=1>, with negligible population of the next excited levels |*S*=2, *M*_*S*_=2> and |*S*=0, *M*_*S*_=0>. This particular field was chosen to maximize the energy separation between the field-induced ground state and the first excited state (see Fig. 2a). The flipping ratios *R* of the most intense reflections were collected (see Supplementary Note 2). A second set of measurements was performed at the same temperature with the sample driven to the |*S*=2, *M*_*S*_=2> ground state with an applied field of 9 T. Magnetization measurements performed on a single crystal of Cr_8_Cd at 1.8 K for a field applied along the *a* axis (Fig. 3) give a value of the magnetization of 2 *μ*_B_ for 4.6 T and 3.8 *μ*_B_ for 9 T, showing that magnetic saturation of the *S*=1 and *S*=2 states, respectively, is practically achieved. The spin density was derived from the flipping ratios by refining the magnetic structure factors using a multipole model^32^ limited to spherical terms. To model the PND data we first used the calculated values of the nuclear structure factors *F*_N_ using the structure determined from the D19 data refinement. In addition, the *F*_N_ values were directly obtained by measuring a selected number of reflections using the same crystal measured on D3. For this purpose, we used the four-circle 5C2 diffractometer at Laboratoire Léon Brillouin (Saclay, France) to measure the integrated intensities at 15 K (see Supplementary Note 2 for more details). The *F*_N_s were calculated as the square roots of the measured intensities with the sign chosen to be the one derived from the structure refinement. The spin densities derived using the measured *F*_N_ are shown in Fig. 4 and the same result is obtained using the *F*_N_ calculated from the structural model. From the data refinement it is found that most of the spin density is located at the Cr position. Allowing spin delocalization on the F and O atoms gives a similar quality fit and the results show that only a small percentage of spin density is delocalized from the Cr on to the neighbour atoms. The experimental values of the spin population are reported in Table 1.

Similar PND studies were performed on a single crystal of the parent closed ring Cr_8_. The experiments were performed using the 5C1 diffractometer at Laboratoire Léon Brillouin. The crystal was mounted with the axis perpendicular to the ring plane making an angle of 90° with the external magnetic field. Flipping ratio measurements were taken for a field of 6 T and at *T*=5 K to populate the first excited *S*=1 state.

## Discussion

**Figure 5:**
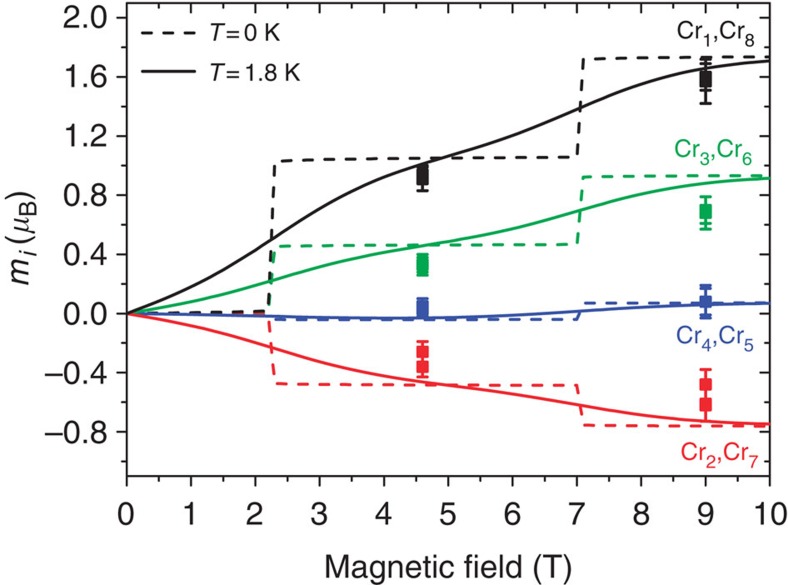
Local spin moments as a function of magnetic field. Calculated local spin moments *m*_*i*_ at the different Cr positions as a function of external magnetic field (lines) as compared to the experimental values (solid circles) for the two measured fields of 4.6 T and 9 T.

The PND experimental results for Cr_8_Cd (Fig. 4) reveal that there is an accumulation of spin density at the edges of the open ring and negligible spin density at the Cr positions further away from the Cd. The sign of the spin moments alternates from positive to negative along the ring, which indicates a staggering of the magnetization. This effect is similar for both 4.6 T and 9 T and increasing the field to 9 T has only the effect of increasing the magnetic moment on each ion, maintaining the same spin distribution. The measured spin moments at different Cr sites are in agreement with the calculations based on the quantum spin Hamiltonian in Equation 1. A comparison of the experimentally determined spin moments with the calculated values using the spin Hamiltonian is shown in Fig. 5. The calculations based on a classical model of an open spin chain by Lounis *et al*.^11^ predict a NC spin arrangement for a chain of eight atoms, which also is in agreement with the values of the projected spin moments along the magnetic field found experimentally.

**Figure 6:**
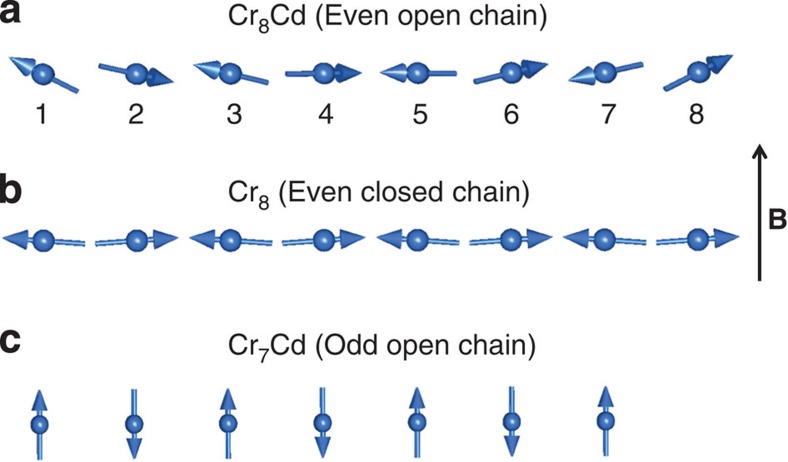
Classical spin ground state configurations. Classical spin ground state configuration for an even-open chain (**a**) an even-closed chain (**b**) and an odd-open chain (**c**) of AF-coupled spins under an external magnetic field.

The classical spin configuration in a magnetic field for an open chain of eight atoms is shown in Fig. 6a. The spins at the extremities of the chain (1 and 8) are more energetically favoured to align along the magnetic field as they have to compete only with one exchange interaction. The AF interaction with the nearest neighbours on sites 2 and 7 would imply that those spins are aligned in the opposite direction. As we move towards the centre of the chain, for the spins at position 4 and 5, the ‘spin-up'—‘spin-down' condition can no longer be satisfied as these two spins cannot simultaneously satisfy the AF condition between them and with their nearest neighbours. This causes frustration and as a result the more energetically favourable ground state is a NC spin configuration.

The situation is very different to that of the Cr_8_ ring. The spins in an even-closed ring under an applied magnetic field are expected to arrange in the SF state (see Fig. 6b)^14^. Hence, the components along **B** of the spin moments are expected to be small and uniform due to the cyclic symmetry of the cluster. The refinement of the PND data for the Cr_8_ compound reveals that the magnetic moment along **B** is only 0.10(2) *μ*_B_ for each magnetic ion, in agreement with the arrangement of the classical spins shown in Fig. 6b. By diagonalizing the microscopic spin Hamiltonian in Equation 1 we obtain a uniform moment of 0.12 *μ*_B_ for *T*=5 K and *B*=6 T, in very good agreement with the measured value. We have therefore demonstrated experimentally the substantial difference between the behaviour of an ‘open' and a ‘closed' even-numbered ring under an applied magnetic field. Our study presents direct quantitative measurement of the local spin moments of the NC and SF states of a finite Heisenberg chain of atoms.

The spin arrangement for the open-even Cr_8_Cd ring is also different to what has been found for the open-odd Cr_7_Cd ring using the NMR technique^33^. In the case of Cr_7_Cd the moments at the different sites are found to be staggered but have a nearly uniform and large value across the ring. This indicates a collinear spin arrangement for an odd-numbered chain Cr_7_Cd (Fig. 6c) as opposed to the NC one for the even-numbered Cr_8_Cd chain. This is also consistent with the fact that for an odd number of ions in a field it is possible to satisfy all the AF spin-up-spin-down arrangements simultaneously and the frustration is relieved. For an odd number of spins the collinear state is the most energetically favourable.

The analysis of the polarized neutron experiments presented here has given quantitative experimental confirmation of the theoretical predictions for the spin arrangement in finite chains of AF-coupled magnetic ions and has shown how the parity of the chain and the boundary condition have a marked effect on the spin structure. This is a general result that can be extended to any short chain of AF-coupled magnetic ions, and we encourage further investigation of odd-numbered closed chains and of integer-spin molecular chains. The latter will provide an unprecedented insight into the spatial structure of their edge states. Furthermore, our conclusions can be extended to the case of chains of AF-coupled atoms deposited on ferromagnetic substrates giving quantitative information on the spin arrangement of such small structures and therefore making essential steps towards the understanding of their suitability in technological applications.

## Methods

### Single crystals preparation

A powder of [H_2_N^*t*^Bu^*is*^Pr][Cr_8_CdF_9_(O_2_CCMe_3_)_18_] (3.35 g) was dissolved in refluxing ethyl acetate (75 ml) in an Erlenmeyer 250-ml flask, while stirring for ≈15 min. The obtained clear solution was allowed to cool to room temperature and left undisturbed at ambient temperature under nitrogen. Large well-shaped crystals were collected after 7 days (typically in the central part of the flask bottom), alongside small crystals (typically at the edge of the flask bottom) including good X-ray quality crystals.

### Magnetization measurements

Magnetization measurements were performed using a Physical Properties Measurement System (Quantum Design) at LaMMB Helmholtz-Zentrum (Berlin, Germany). A single crystal of (1 × 1 × 2.3) mm^3^ was mounted with the crystallographic *a* axis along the magnetic field. Measurements were performed with the sample at 1.8 K and magnetic fields up to 14 T.

### Neutron diffraction

The 15 K neutron data collection was performed on the thermal four-circle D19 diffractometer equipped with a very large (120° × 30°) position-sensitive detector, at the ILL (Grenoble, France), using an incident wavelength of *λ*=1.4587 Å. A single crystal of size (1.6 × 1.6 × 7) mm^3^ was mounted in a thin-walled quartz tube and a two-stage Displex cryorefrigerator was used to cool the sample to 15 K at a rate of 2 K per min.

### Polarized neutron diffraction

A larger (2.2 × 2.3 × 7) mm^3^ single crystal with respect to the one used on D19 was used for the PND measurements. Flipping ratio measurements were performed on the D3 polarized-beam diffractometer at the ILL with an incident wavelength of 0.825 Å. The beam polarization was equal to 0.95(1). The sample was sealed in a thin-walled quartz tube and cooled slowly (2 K per min) in zero-field from 300 K to 1.8 K. There are four wheels in the unit cell each making an angle of 75.5° between the perpendicular to the mean plane of each wheel and the *a* axis. The orientation of the crystal was chosen with the *a* axis vertical, along the magnetic field. This was to induce equal components of the magnetic moments along *a* for the four molecules in the unit cell. The orientation of the crystal in the cryomagnet was refined using the P2_1_/n space group.

## Author contributions

T.G, B.G., S.A.M., A.S., J.v.S. and A.C. performed the neutron experiments, T.G., B.G., S.A.M. and R.C. performed the data treatment and analysis, B.K. and T.G. performed the magnetization measurements, E.G., S.C. and P.S. performed the numerical simulations and modelled the experimental results, G.A.T. developed the chemical strategy with R.E.P.W. and synthesized the crystals, T.G. wrote the paper with the input from all the co-authors.

## Additional information

**How to cite this article**: Guidi, T. *et al*. Direct observation of finite size effects in chains of antiferromagnetically coupled spins. *Nat. Commun*. 6:7061 doi: 10.1038/ncomms8061 (2015).

## Supplementary Material

Supplementary Information Supplementary Table 1, Supplementary Notes 1-2 and Supplementary References

## Figures and Tables

**Table 1:** Results of the spin density refinement.

*Refinement details*		
Magnetic field	4.6 T	9 T
Spin state	*S*=1	*S*=2
*N*_o_ with |1−*R*|>*σ*	187	126
*N*_v_	9	13
GOF	1.38	1.9
*R*_w_(|1−*R*|)	0.17	0.13
		
*Local magnetic moments m*_i_ (*μ*_B_)		
Cr1	0.91(4)	1.57(8)
Cr2	−0.26(4)	−0.48(5)
Cr3	0.33(4)	0.68(6)
Cr4	0.04(3)	0.08(6)
Cr5	0.03(3)	0.08(5)
Cr6	0.33(3)	0.70(5)
Cr7	−0.36(4)	−0.62(6)
Cr8	0.94(3)	1.60(5)
Sum	1.96(9)	3.61(16)

*σ*, experimental error bar on the flipping ratio *R*(hkl), *N*_o_, number of observed flipping ratios with |1−*R*|>*σ, N*_v_, number of varied parameters; GOF, goodness of fit; *R*_w_, weighted agreement factor; weighting scheme: *w*=1/*σ*^2^.
