# Supplementary material for: Direct observation of finite size effects in chains of antiferromagnetically coupled spins
Source: Nat Commun. 2015 May 8;6:7061. doi: 10.1038/ncomms8061 (PMC4432630; doi:10.1038/ncomms8061)
Supplement: Supplementary Information — Supplementary Table 1, Supplementary Notes 1-2 and Supplementary References [file ncomms8061-s1.pdf]

Supplementary Table 1:

Crystallographic parameters for Cr_8Cd at 15 K

	**Crystal data**	
**Formula**	$Cr_8Cd_1F_9C_{90}H_{180}O_{36}N$	
**Formula weight**	2635.82	
**Crystal system**	Monoclinic	
**Color**	dark green	
	**Neutron data collection**	
**Instrument**	D19	
**T(K)**	15	
**Crystal size (mm^3)**	$1.6\times 1.6 \times 7$	
	**Refinemen Details**	
**Space group**	P 2_1/n	P 2_1/c
**a (Å)**	19.452(1)	35.485(3)
**b (Å)**	22.198(1)	22.177(2)
**c (Å)**	30.653(2)	37.081(2)
β **(degrees)**	92.806(3)	115.243(4)
**Volume (Å^3)**	13220	26394.7
**Z**	4	4
**N reflection used**	13905	21493
**R(F) $F_o \geq 4\sigma(F_o)$**	0.28	0.24

R=agreement factor; R(F)=$\sum \| |F_O| - |F_C| \| / \sum |F_O|$; F_O=observed structure factor; F_C=calculated

structure factor

Supplementary Note 1

Low temperature structure determination (15 K)

A previous X-ray diffraction study at 100 K [1] showed that the Cr_8Cd compound crystallizes in the monoclinic $P2_1/n$ space group, with a = 19.3987(8) Å, b = 22.2606(8) Å, c = 31.3094(14) Å, β = 91.663(4) degrees and Z = 4. To determine the low temperature structure we used the thermal four-circle D19 diffractometer at the ILL. At low temperature (15 K), weak extra reflections were observed out of the peak positions corresponding to the $P2_1/n$ space group. The cell parameters refined on 4032 indexed reflections are : a = 19.452(1) Å, b = 22.198(1) Å, c = 30.653(2) Å, β = 92.806(3)°. The refinement of the structure with the SHELX97 program [2] using the X-ray structure at 100 K as initial model on 13905 reflections (with observed structure factor $F_O \geq 4\sigma(F_O)$) indexed in the $P2_1/n$ group provided an agreement factor R of 0.28 (Supplementary Table S1) and a goodness of fit S = 2.38. Disorder is observed on most of the -C(Me)$_3$ groups of the pivalate ligands. In order to better understand the low temperature structure, a structural study was performed afterwards by X-ray diffraction at 30 K at Durham University on a Cr_8Cd crystal coming from the same batch of crystals as the one measured on D19. It was found that all reflections could be indexed in the $P2_1/c$ group, with a doubling of the previous unit cell and two different molecules in the asymmetric unit instead of one for the $P2_1/n$ group. The two molecules differ mostly by the positions of the pivalate atoms linked to Cr atoms whilst the positions of the Cr and Cd atoms are essentially not affected. The neutron data collected on D19 were then re-analyzed in

the $P2_1/c$ space group. The following cell parameters were obtained at 15 K by refinement on 3692 reflections: *a* = 35.485(3) Å, *b* = 22.177(2) Å, *c* = 37.081(2)Å, β = 115.243(4)°. Starting from the X-ray structure at 30 K, the refinement on 21493 reflections ($F_o > 4\sigma$) leads to an agreement factor *R(F)* = 0.24 (0.35 for all 48314 data) for 3086 refined parameters. Further X-ray diffraction measurements as function of *T* showed that the transition temperature is about 165 K.

Supplementary Note 2

Polarized Neutron diffraction experiment

Polarized neutron diffraction measurements were performed on the D3 polarized-beam diffractometer at the ILL (Grenoble). At 1.8 K and under magnetic field of 4.6 T, the flipping ratios of the most intense reflections were collected, in order to investigate the $| S$=1,M_S=1$>$ ground state, leading to a set of 187 reflections with ($|1 - R| > \sigma$) including (h,k,l) and (h,-k,l) equivalent reflections. A second set of measurements was performed at the same temperature with the sample driven to the $| S$=2,M_S=2$>$ ground state with an applied field of 9 T. A set of 126 reflections with ($|1 - R| > \sigma$) was obtained. Moreover, in order to verify that the flipping ratios are not affected by structural disorder, that could be different for two different crystals or cooling procedure, flipping ratio measurements were repeated on the polarized neutron diffractometer 6T2 at LLB on the small single crystal. Comparison of the flipping ratios showed very good agreement between the two sets of data collected on the large crystal on D3 and the small crystal on 6T2. In order to determine the nuclear structure factors at low temperature on the same large crystal as the flipping ratios, integrated intensities were measured at 15 K on the 4-circle 5C2 at LLB for a set of 279 reflections corresponding to the list of reflections that were measured on the polarized neutron diffractometer

D3 with the indexing corresponding to the small unit cell. A total of 794 reflections were collected which provided a set of 124 unique reflections, after absorption correction, including Friedel reflections. The absorption coefficient was determined by transmission measurement of the direct neutron beam through the crystal: μ = 0.546 cm^{-1}.

Supplementary References

1. Timco, G. A. *et al.* Influencing the nuclearity and constitution of heterometallic rings *via* templates. *Chem. Commun.* 3649–3651 (2005).

2. SHELXL97. *G. M. Sheldrick, University of Göttingen* (1997).
